# Effects of Tobacco on the Mental Powers

**Published:** 1866-08

**Authors:** 


Effects of Tobacco on the Mental Powers.—M. Bertillon, in the *Union Medicale*, supplies some interesting facts in relation to the effects of tobacco on the mental powers, derived from an investigation made at the Polytechnic School in 1855-56. He investigated in 160 of the pupils who had undergone their examination, what influence the fact of their being smokers had upon the result. As large a proportion as 102 of these pupils were smokers. It was found that in the classification by merit which followed the examinations, that, while in the highest series a third or fourth of the pupils were smokers, in the lower three-fourths, and in the lowest series four-fifths were smokers. Again, while among 66 confirmed smokers their mean rank of 94.6 on their entrance into the school had sunk to 98.3, in the case of the 60 pupils who were not smokers their rank of 71 on entrance (already 23 ahead of the smokers) rose to 67.5—being, as the result nine months' work in common, 30 in advance of the smokers. This result of the inquiry as regards these limited numbers was conformable to the prior experience of the school—*Medical and Surgical Reporter.*

